# Potentiometric performance of flexible pH sensor based on polyaniline nanofiber arrays

**DOI:** 10.1186/s40580-019-0179-0

**Published:** 2019-03-18

**Authors:** Hong Jun Park, Jo Hee Yoon, Kyoung G. Lee, Bong Gill Choi

**Affiliations:** 1 Department of Chemical Engineering, Kangwon National University, 346 Joongang-ro, Samcheok, Gangwon-do 25913 Republic of Korea; 2 Nano-Bio Application Team, National NanoFab Center (NNFC), Daejeon, 34141 Republic of Korea

**Keywords:** Screen printing, pH sensor, Electrochemistry, Polyaniline, Nanofiber

## Abstract

We report potentiometric performance of a polyaniline nanofiber array-based pH sensor fabricated by combining a dilute chemical polymerization and low-cost and simple screen printing process. The pH sensor had a two-electrode configuration consisting of polyaniline nanofiber array sensing electrode and Ag/AgCl reference electrode. Measurement of electromotive force between sensing and reference electrodes provided various electrochemical properties of pH sensors. The pH sensor show excellent sensor performances of sensitivity of 62.4 mV/pH, repeatability of 97.9% retention, response time of 12.8 s, and durability of 3.0 mV/h. The pH sensor could also measure pH changes as the milk is spoiled, which is similar to those of a commercial pH meter. The pH sensors were highly flexible, and thus can measure the fruit decay on the curved surface of an apple. This flexible and miniature pH sensor opens new opportunities for monitoring of water, product process, human health, and chemical (or bio) reactions even using small volumes of samples.

## Introduction

Detecting pH value, commonly measuring acidity and alkalinity in a given solution, can be used as an indicator for assessment of environment, biochemical, and biological processes [1−4]. The pH sensing techniques are based on various methods, such as, potentiometric, capacitive, chemiresistive, luminescence, sand optical methods [5–8]. Potentiometric pH sensor is the most popular among the pH sensors as its size can be reduced, device structure is simple, and unit cost of fabrication is relatively low [5]. The potentiometric pH sensor measures the difference in electromotive force (EMF) between a pH sensing electrode and a reference electrode. As a reference electrode, an Ag/AgCl is the most commonly used because of its potential stability and environment friendly. The common pH sensing electrodes involve metal oxides (e.g., IrO_x_, WO_3_, RuO_2_, TaO_2_, TiO_2_, and SnO_2_) and conducting polymers [9–16]. As application of pH sensor expands ranging from laboratory experimentation to agriculture, water quality, healthcare, and clinical applications, advances in pH sensors have been miniaturized and flexible, enabling direct measurement of pH in limited volume or curved surfaces. In addition, many applications require reduction of unit cost of pH sensor fabrication.

Printing technologies have recently gained a great deal of attention for fabrication of electronics and electrochemical devices, including displays, sensors, energy devices, and electronic devices [17–20]. Screen printing, inkjet printing, and photolithography have been developed for printed electronics and electrochemical devices. A screen printing is based on a mask (screen) to transfer pastes onto a substrate, excluding the areas made impermeable to the paste by a blocking stencil [21–23]. This is a scalable and low-cost process and is suitable for micro-sized materials and devices.

Herein, we report a screen printing process for fabricating flexible and miniature pH sensors. The pH sensor has two-electrode configuration consisted of a polyaniline nanofiber array-based sensing electrode and an Ag/AgCl reference electrode. The pH sensor is based on potentiometric technique, measuring EMF between two electrodes. The pH sensor accurately and quantitatively measured pH levels of samples according to a Nernstian behavior. The pH sensor showed excellent sensor performances of sensitivity of 62.4 mV/pH, repeatability of 97.9% retention, response time of 12.8 s, and durability of 3.0 mV/h. Moreover, the pH sensor could measure pH changes of food spoilage, such as, a milk and an apple. The flexibility of pH sensor enabled to measure pH level on the curved apple surface.

## Results and discussion

**Fig. 1:**
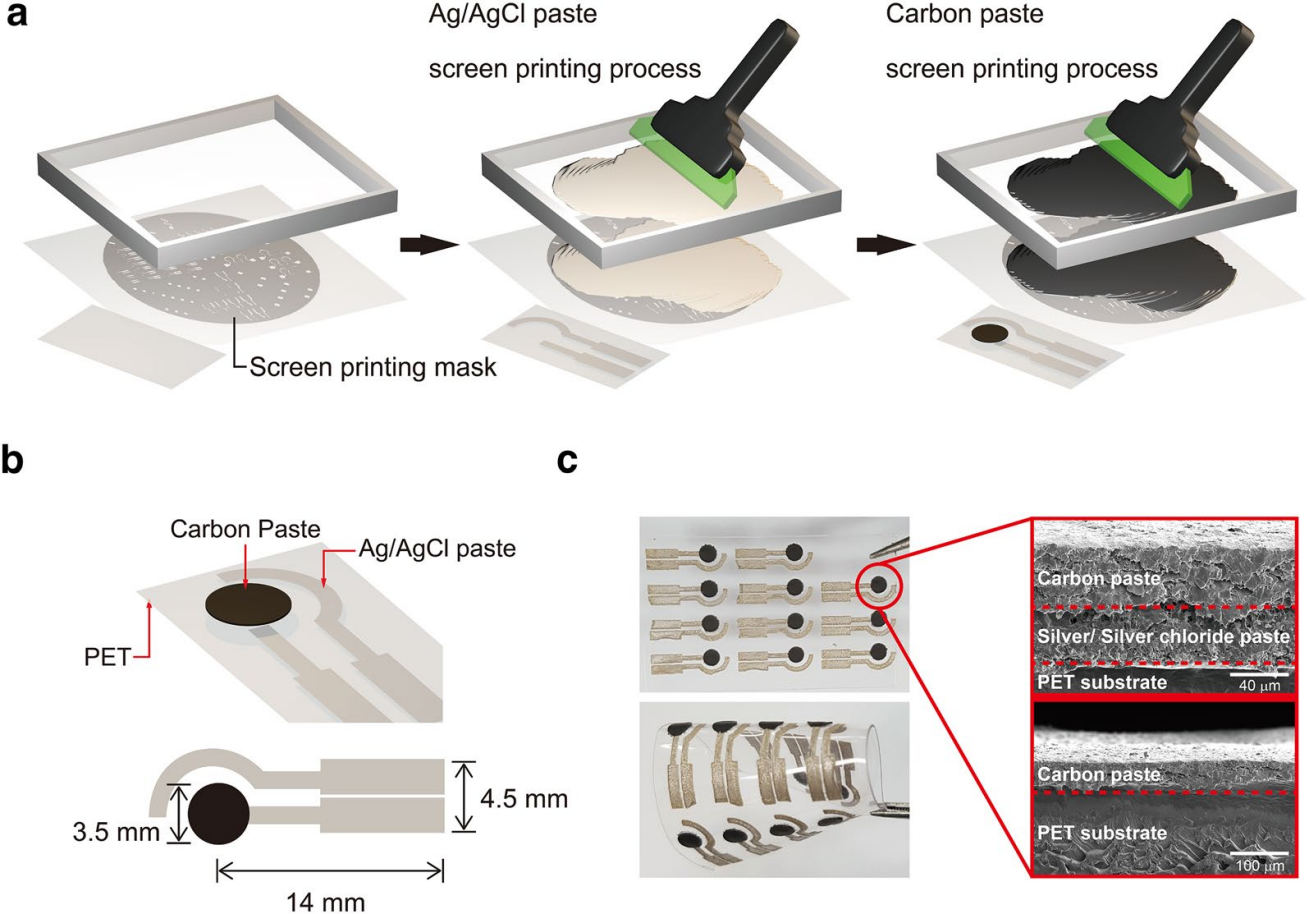
**a** Schematic illustration of fabrication of pH sensors based on a screen printing process. **b** Schematic of device structure of pH sensor. **c** Photograph images of pH sensors under mechanically bent and normal states and cross-sectional SEM images of pH sensors

Figure 1a shows the experimental procedure of pH sensor based on screen printing process using carbon and Ag/AgCl pastes. The pH sensor has a two-electrode configuration with a dimension of 17.5 × 4.5 mm^2^ (Fig. 1b). The sensing electrode has a circle shape and semi-circle shape is for a reference electrode. As a conductive circuit, an Ag/AgCl paste was printed onto a polyethylene terephthalate (PET) substrate. The pre-patterned stainless steel mask transferred Ag/AgCl paste onto a substrate. The Ag/AgCl was strongly attached onto the surface of PET. As for working electrode, carbon paste was printed onto Ag/AgCl electrode using a screen printing process. When observing interface between carbon and Ag/AgCl electrodes using SEM measurement, there is no cracks or holes, indicating strong adhesion of carbon and Ag/AgCl electrodes (Fig. 1c). The screen-printed pH sensors can be easily flexible. This screen printing process enabled to a scalable fabrication of pH sensors with a low-cost and high-throughput process (Fig. 1c).

**Fig. 2:**
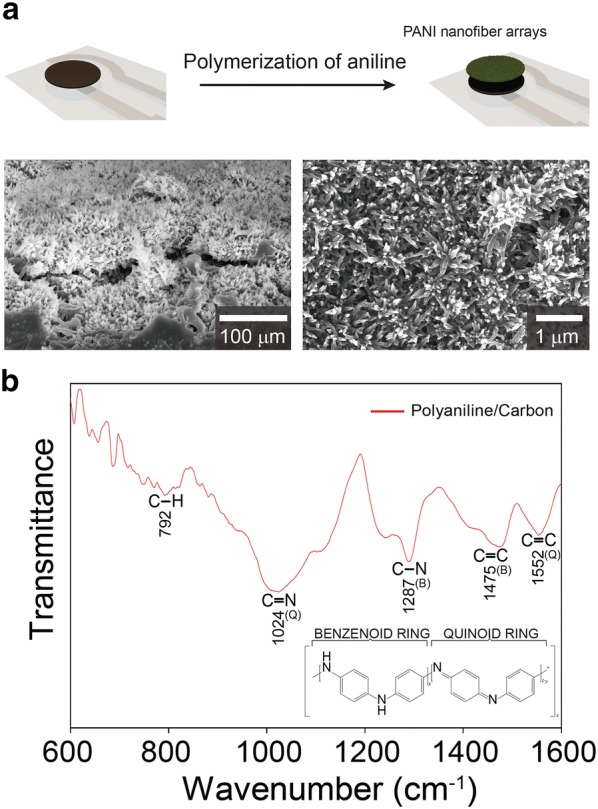
**a** SEM images of PANI nanofiber arrays on pH sensor with schematic illustration of PANI polymerization procedure. **b** FT-IR spectrum of PANI coated on carbon electrode

To complete sensing electrode, polyaniline (PANI) nanofiber arrays were deposited onto the surface of carbon electrode by a dilute chemical polymerization method according to the previous reports [24]. The ordered PANI nanofiber arrays covered the smooth surface of carbon pastes (Fig. 2a), resulting in a rough morphology with a high surface area. The PANI-based electrodes are highly sensitive to H_3_O^+^ ions, and their redox equilibrium between H_3_O^+^ and PANI phase transitions is suitable for potentiometric pH sensors [15, 16]. The nanostructure and high specific surface area of PANI electrode enable to facilitate redox reactions, and thus leading to potential stability of pH sensors [15]. In addition, PANI is low-cost, biocompatible, and reproducible. The successful synthesis of PANI was demonstrated by FT-IR spectroscopy (Fig. 2b). The prominent peaks of PANI-based electrode at 1552, 1475, 1287, 1024 and 792/cm correspond to the C=C stretching of the quinoid ring, C=C stretching of the benzenoid ring, the C‒N stretching of the benzenoid unit, the C‒N stretching of quinoid unit, and the C‒H out-of-plane bending [25–27].

**Fig. 3:**
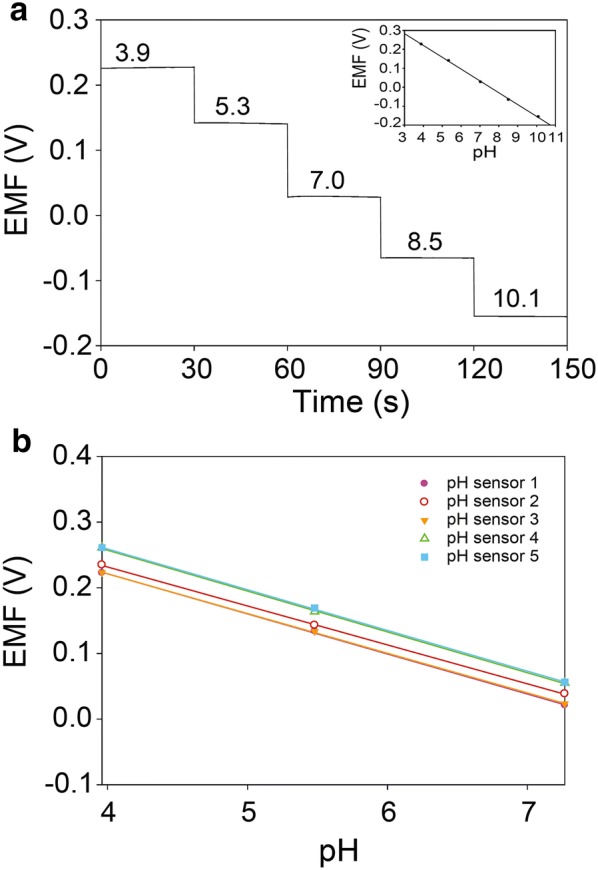
**a** EMF responses of pH sensor measured in buffered solutions of pH 3.9‒10.1. Inset is a plot of EMF signals versus pH. **b** Reproducibility test of pH sensor measured in buffered solution of pH 3.9, 5.5, and 7.3

**Fig. 4:**
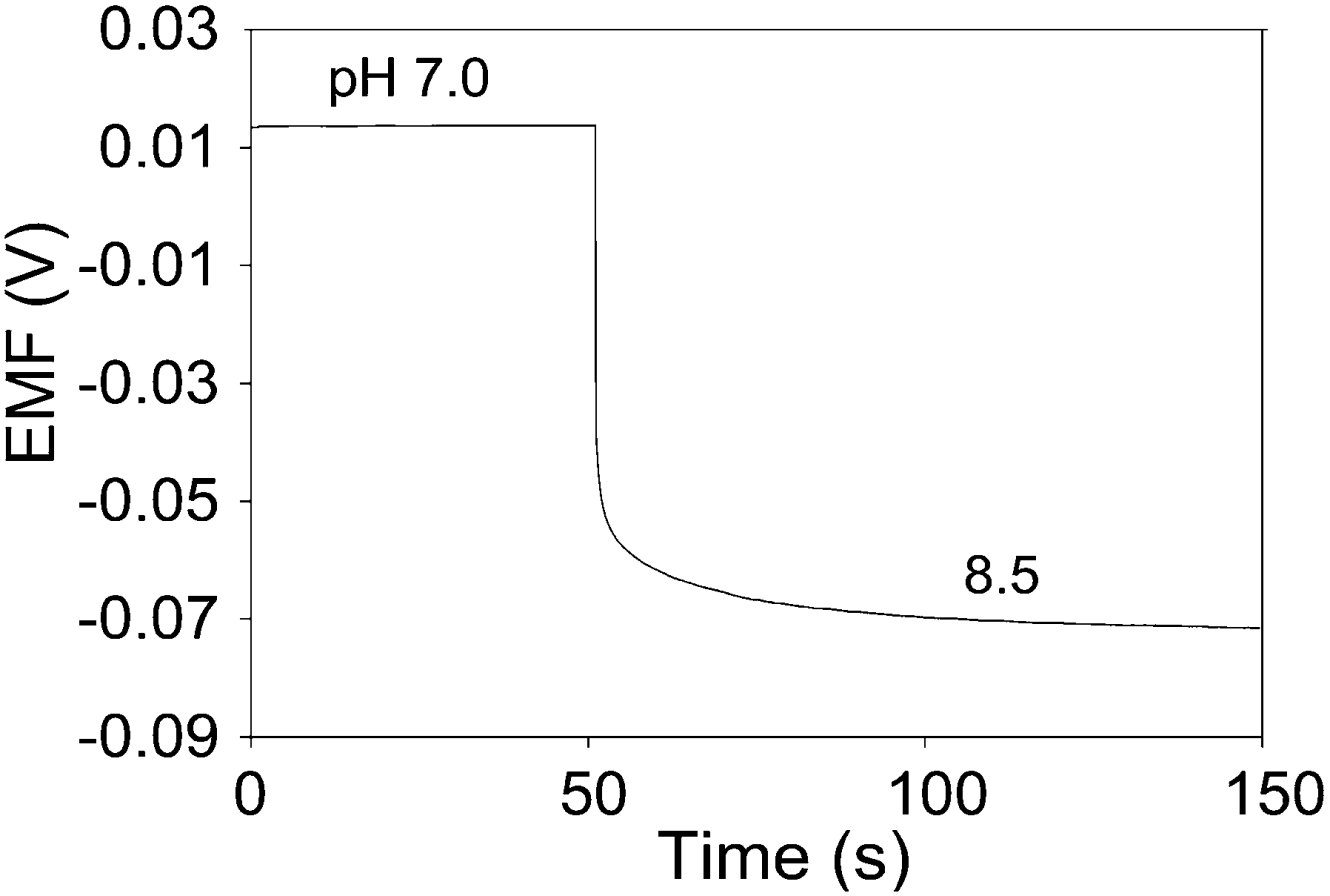
Temporal EMF response in titration from pH 7.0 to pH 8.5 for pH sensor

The measurement of EMF between PANI sensing and Ag/AgCl reference electrodes provided electrochemical characteristics of pH sensor. The EMF signals of pH sensor were obtained by varying pH levels ranging from 3.9 to 10.1 (Fig. 3a). The pH values of buffered solution were controlled by adding HCl or NaOH solution into buffered solutions. In order to ensure accurate measurement of pH value of buffered solution, a commercial pH meter (ORION™ Star A211) validated the pH values. When changing pH value of buffered solution, EMF signals dramatically changed, and then spontaneously reached a steady state. EMF signals were collected from steady state values. Inset of Fig. 3a shows a plot of EMF versus pH levels, resulting in a standard calibration line for pH sensors. The sensitivity of potentiometric pH sensor can be obtained by the slope of the linear regression according to the Nernstian equation [6]:

1 $$E = E^{0} - \left( {2.303RT/F} \right){\text{pH = }}E^{0} - 0.05916\;{\text{pH}}$$

where *E*^0^ is the standard electrode potential, *R* is the gas constant, *T* is the temperature, and *F* is Faraday’s constant. Based on Nernstian behavior, the theoretical maximum sensitivity is –59 mV/pH at room temperature. The resultant calibration curve is linear with a slope of 62.4 mV/pH (R^2^ = 0.9984) in a wide pH range of 3.9–10.1. This sensitivity value (62.4 mV/pH) of pH sensor is close to the theoretical pH sensitivity based on Nernstian behavior and is similar to other previous reports based on PANI electrodes [28–31]. The screen-printing-based fabrication method of pH sensors provided a good reproducibility for pH sensors (Fig. 3b). Five samples of pH sensors were tested, resulting in an average sensitivity of 60.4 ± 1.1 mV/pH and average standard electrode potential of 479.6 ± 20.1 mV. The response time of pH sensor was measured by increasing pH level from 7.0 to 8.5 (Fig. 4). The EMF signal was changed subsequently and reached 90% of its steady state value within 12.8 s. This fast response time of pH sensor indicates that pH sensor enable to monitor dynamic changes of analyte.

**Fig. 5:**
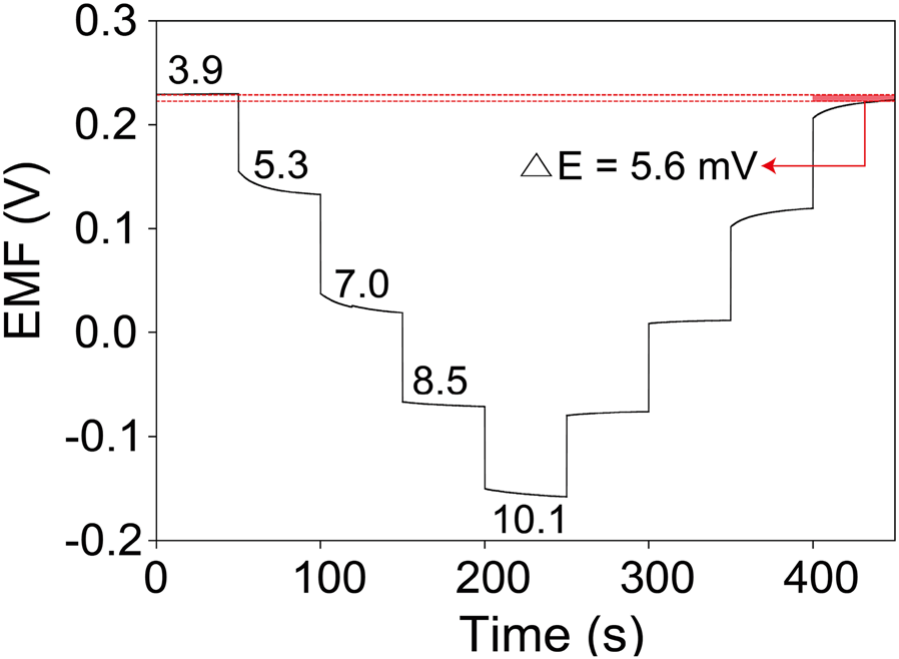
Repeatability test of pH sensor with varying pH levels in a range of 3.9‒10.1

The repeatability is essential for high-performance pH sensor. The pH sensor should endure to long periods under oscillating pH changes. The repeatability of pH sensor was investigated by measuring EMF responses of pH sensor during a titrated-cycle in pH 3.89, 5.34, 7.03, 8.51, and 10.09 (Fig. 5). During this titration process, 1M HCl or NaOH solution was dropped into buffered solution to decrease or increase the pH levels of buffered solution. The pH values were validated with a commercial pH meter (ORION™ Star A211). The pH sensor was continuously tested without washing step or other treatment. Reading the pH value was performed after 15 s of titration step. The initial pH sensitivity is 62.9 mV/pH (R^2^ = 0.9982), and this value is slightly decreased after titration process (61.6 mV/pH, R^2^ = 0.9964). This indicates an excellent repeatability performance. As the usage of pH sensor increases, the electrochemical potential of pH sensor can be influenced. This phenomenon is referred to as hysteresis, which is related to the reversibility. After titration process, the initial electrochemical potential (229.7 mV) of pH sensor at pH 3.89 decreased to 224.1 mV with a small hysteresis width of 5.6 mV, indicating no memory effect of hysteresis of pH sensor.

**Fig. 6:**
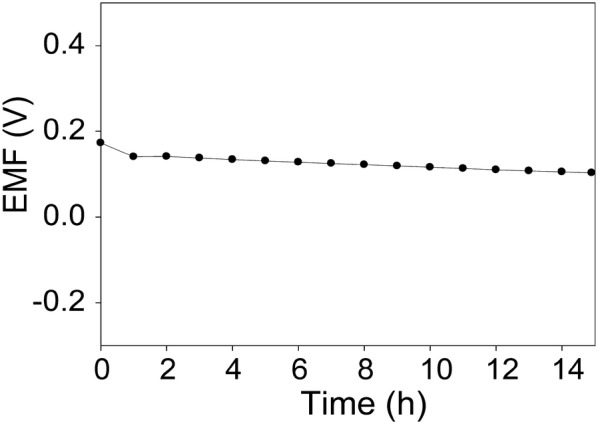
Long-term stability of pH sensors measured in a buffered solution at pH 5.5

One of the most important factors for potentiometric pH sensors is ability to selectively detect and measure H_3_O^+^ ion in the presence of other ions. In this study, we adopted IUPAC recommended separate-solution method (SSM) to evaluate selectivity coefficients of pH sensors against different interfering ions of Ca^2+^, Mg^2+^, K^+^, Na^+^, and NH_4_^+^. The EMF responses were measured in a mixture containing differeent cations at a same concentration of 10^−2^ M. The selectivity coefficient (K) in SSM can be defined as follows [15, 32]:

2 $$K_{IJ}^{POT}$$

where *I* is primary ion, *J* is interfering ion, and *POT* is potential. The measured K values of pH sensor are shown in Table 1. All of the K values for pH sensor are below 10^−9^. IUPAC indicates that if the pH sensor has a K value < 1, the pH sensor can accurately measure H^+^ over interfering ions. Based on these results, pH sensor fabricated in this work demonstrated an excellent ion selectivity. The long-term stability of pH sensor was further investigated by measuring EMF responses of pH sensors immersed in pH 5.5 over 15 h (Fig. 6). The pH sensor maintained EMF signals during 5‒12 h, resulting in a potential drift of 3.0 mV/h, which corresponds to 4.8% error in pH value over an hour of continuous measurement.

**Table 1:** Selective coefficients of pH sensors using SSM for primary ion (H^+^) against interfering ions

Ions (J)	log K_IJ_^POT^	K_IJ_^POT^
Na^+^	− 9.80	1.59 × 10^−10^
K^+^	− 8.86	1.37 × 10^−9^
NH_4_^+^	− 10.23	5.82 × 10^−11^
Ca^2+^	− 9.56	2.70 × 10^−10^
Mg^2+^	− 11.48	3.34 × 10^−12^

**Fig. 7:**
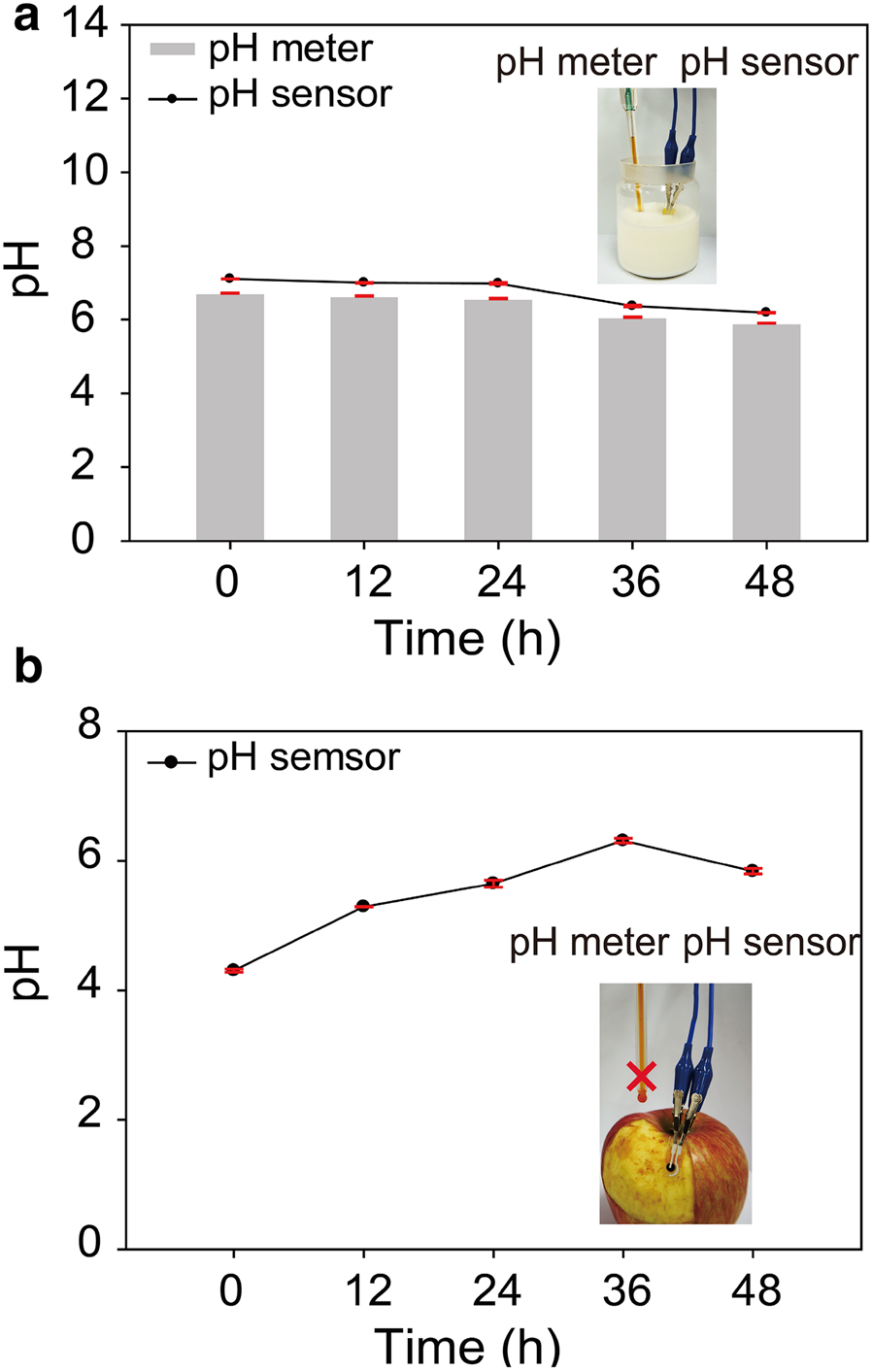
**a** Milk and **b** apple spoilage measurements using pH sensor and pH meter (OrionTM Star A211)

Detecting the quality of the foods has been attracted much attention in modern human safety and food product markets [32–35]. The pH sensors could provide an desirable spoilage indicator for food quality. Various bacteria species were grown on food under certain conditions, resulting in food spoils. The growth of many organisms and bacteria varies pH level of food. We monitored milk and apple spoilages using a pH sensor (Fig. 7). The milk and apple were conditioned at 35 °C and humidity of 85%. The pH of fresh milk was approximately 6.7. As milk spoils, the acidity increases because the lactic acid bacteria grow and produce lactic acid [36]. During 48 h, the pH decreased from 7.1 to 6.2, indicating milk spoilage (Fig. 7a). These pH changes were similar to those of a commercial pH meter (ORION™ Star A211). The pH sensor can be also quantified to measure apple quality (Fig. 7b). The monitoring of pH change was performed on the curved apple surface using a flexible pH sensor. The commercial pH meter cannot be used in this experiment. The unspoiled apple shows strong acidity of pH 3.8. The bacterial spoilage increased pH of an apple surface (pH 5.9) after 48 h.

## Conclusions

We developed a highly flexible pH sensor based on a screen printing process using a carbon and Ag/AgCl pastes. This screen printing process provided a low-cost, simple, and scalable fabrication of pH sensors. As a flexible sensing electrode material, polyaniline nanofiber arrays were deposited on carbon-working electrode by a dilute chemical polymerization. The electrochemical properties of pH sensor were characterized for sensitivity, response time, repeatability, and stability. The sensitivity (62.4 mV/pH) of pH sensor was close to theoretical value based on Nernstian behavior. Moreover, the pH sensor showed an excellent response time of 12.8 s, repeatability of 97.9% retention, and stability (drift of 3.0 mV/h). To demonstrate practical application, the pH sensors measured a disagreeable change in a normal state of food. The pH change of milk spoilage is similar to a commercial pH meter. In addition, the flexible pH sensor measured pH changes of food spoilage on the curved apple surface, which cannot be measured using a commercial pH meter. The flexible pH sensor based on a screen printing process could be applied to many applications, including monitoring of water, product process, human health, and chemical (or bio) reactions.

## Experimental

### Materials

Potassium hydrogen phthalate, potassium phosphate monobasic, tris(hydroxymethyl)aminomethane, aniline (99.5%), ammonium persulfate, sodium hydroxide, hydrochloric acid, calcium chloride, magnesium chloride, ammonium chloride, potassium chloride, methanol and butvar® B-98 were purchased from Sigma-Aldrich (USA). Borax anhydrous was purchased from Fluka (UK). Sodium chloride was purchased from Junsei (Japan). Sulfuric acid was purchased from Showa (Japan). Carbon paste (7105 conductor) was purchased from Dupont (USA). Silver/silver chloride was purchased from ERCON (USA). Deionized (DI) water (18.2 MΩ/cm) was employed in all experiment.

### Preparation of electrical electrodes using screen printing process

The carbon and silver electrodes were fabricated using a screen printing coater (MTI Korea, EQ-SPC-2) and custom stainless steel stencils developed using AutoCAD software. A flexible polyethylene terephthalate (PET) substrate was used. An Ag/AgCl paste was printed according to the electrode patterns. The Ag/AgCl electrode was used as a reference electrode. The printed electrodes were cured at 85 °C for 20 min under vacuum. The working electrode was prepared by screen printing of carbon paste with a circle shape in a 3.5 mm diameter. After printing, the electrode was cured at 85^o^C for 20 min.

### Fabrication of pH sensor

To complete pH sensor fabrication, polyaniline (PANI) was synthesized onto the surface of carbon through a dilute chemical polymerization according to previously reported method [24]. The printed electrodes were immersed in a mixture of aniline monomer (10 mM) and ammonium persulfate (6.7 mM) in perchloric acid (1 M, 100 mL), and was stirred at approximately 4 °C for 24 h. An Ag/AgCl electrode was covered with polyvinyl butyral (PVB) containing NaCl. The resultant pH sensors were dried at room temperature under vacuum.

### Characterization

Scanning electron microscopy (SEM) images were obtained using a field emission scanning electron microscope (S-4800). Fourier transform infrared (FT-IR) spectra were collected on a JASCO FT-IR 4600. Each spectrum was recorded from 4000 to 400/cm. Electrochemical characterization was performed using a CHI 760E (CH instrument, Inc.). All electrochemical measurements were performed at room temperature, and the obtained data were within the error range of ± 1%. Sensitivity, repeatability, response time, selectivity, and stability test were performed using a potentiometric technique.

## Abbreviations

EMF: electromotive force
SSM: separate-solution method
PET: polyethylene terephthalate
PANI: polyaniline
PVB: polyvinyl butyral
SEM: scanning electron microscopy
FT-IR: Fourier transform infrared
